# Development and preliminary validation of a novel eating disorder screening tool for vegetarians and vegans: the V-EDS

**DOI:** 10.1186/s40337-024-00964-7

**Published:** 2024-01-09

**Authors:** Courtney P. McLean, Zhibin Chen, Richard Song, Jessica Le, Joanne Fielding, Gemma Sharp

**Affiliations:** 1 Department of Neuroscience, Monash University, 99 Commercial Rd, Melbourne, VIC Australia; 2 University of Melbourne, Grattan Street, Parkville, VIC Australia; 3 Alfred Hospital, 55 Commercial Rd, Melbourne, VIC Australia

**Keywords:** V-EDS, Eating disorder, Screening tool, Vegetarian, Vegan, Test development, Validation

## Abstract

**Background:**

Eating disorders have one of the highest mortality of all mental illnesses but are associated with low rates of screening and early intervention. In addition, there remains considerable uncertainty regarding the use of current standardised screening tools in measuring eating pathology in vegetarians and vegans. With these groups presenting as potential at-risk groups for disordered eating development, the present study aimed to develop and preliminary validate a novel eating disorder screening tool, the Vegetarian Vegan Eating Disorder Screener (V-EDS).

**Methods:**

We utilised a mixed-methods approach, comprising four phases.

**Results:**

A conceptual framework was developed from 25 community, clinician, and lived experience interviews and used to derive a preliminary set of 163 items (Phase 1). Phase 2 piloted the items to establish face and content validity through cognitive debriefing interviews of 18 additional community, clinician, and lived experience participants, resulting in a reduced, revised questionnaire of 53 items. Phase 3 involved scale purification using Item Response Theory in analysis of 230 vegetarians and 230 vegans resulting in a further reduced 18-item questionnaire. Phase 4 validated the screening tool in a large community sample of 245 vegetarians and 405 vegans using traditional psychometric analysis, finding the V-EDS supports a unidimensional factor structure with excellent internal consistency (α = 0.95–0.96) and convergent validity (0.87–0.88), and moderate discriminate validity (0.45–0.55).

**Conclusions:**

This study provided strong initial support for the psychometric validity and theoretical assumptions of the novel V-EDS screening tool. The V-EDS has the potential to increase early intervention rates for vegetarians and vegans experiencing eating disorder symptoms, further supporting advocacy and treatment approaches for these expanding dietary groups.

**Supplementary Information:**

The online version contains supplementary material available at 10.1186/s40337-024-00964-7.

## Abstract (plain language summary)

The present study describes the development and preliminary validation of the first screening tool designed to uniquely assess eating disorder symptoms in individuals following a vegetarian or vegan diet. Following several development phases, the final version of the Vegetarian Vegan Eating Disorder Screener (V-EDS) comprises 18-items, with six dietary characteristic items and 12 eating disorder scored items. The current findings support excellent initial reliability and validity of the V-EDS. The V-EDS constitutes a promising tool that could potentially be integrated as a standalone measure for initial screening in clinical and research settings, but also for more comprehensive assessment when combined with other gold-standard eating disorder tools.

**Supplementary Information:**

The online version contains supplementary material available at 10.1186/s40337-024-00964-7.

## Background

The number of people following a vegetarian or vegan diet is increasing around the globe, with estimates suggesting approximately 12% and 2% of the Australian population are vegetarian or vegan, respectively [1, 2]. These rates have been similarly demonstrated in other Western countries such as the United Kingdom and Germany [3, 4], and have been noted to be driven by a variety of reasons such as increasing animal welfare and environmental sustainability concerns, and the positive health benefits of consuming more plant-based foods [5, 6]. Within the eating disorder research field however, vegetarianism and veganism have long been thought to be related to an elevated risk of eating disorder symptoms [7–10]. For example, a degree of dietary restraint is necessary for vegetarian and vegan diets in order to consciously regulate the consumption of meat and/or animal products [11, 12], though this may not necessarily be driven by weight or shape control reasons (i.e., cognitive restraint; defined as the limited dietary intake to manage body weight). Yet indeed, the distinction between dietary restraint and cognitive restraint is often blurred within the vegetarian/vegan literature, positing that vegetarianism and veganism may act as a socially acceptable way to restrict food intake for weight control means and conceal disordered eating behaviours [13–16]. A systematic review of 48 studies examining the link between vegetarianism, veganism, and higher levels of disordered eating were unable to confirm this association with overall mixed findings [11]. The authors also noted that the majority of included studies had satisfactory to poor quality ratings, indicating a potential high risk of bias due to poor reporting of methodological and participant characteristics. For example, vegetarian and vegan samples were often disproportionately small relative to omnivore control groups or frequently grouped into one sample meaning that true associations between groups may have been masked.

The identification of eating disorders can be achieved through clinician-led interviews, such as the widely used Eating Disorder Examination Interview (EDE; [17]), or self-report questionnaires which are most commonly employed as screening or assessment tools. However, literature to date examining the use of eating disorder tools in strict vegetarian and vegan populations is scarce and focuses largely on the employment of self-report questionnaires [11, 18]. For example, Heiss, Boswell [19] were unable to support the original and alternate factor models of the gold-standard eating disorder tool, the Eating Disorder Examination-Questionnaire (EDE-Q; [20]) in a sample of 318 vegans, and further validated in separate analyses of vegetarians and vegans [6]. More positive findings have been reported for brief versions of the EDE-Q, with Zickgraf et al. [21] demonstrating strict measurement invariance of the Short-EDE-Q between non-vegetarians/vegans, weight-motivated vegetarians/vegans, and non-weight-motivated vegetarians/vegan university students. Heiss et al. [22] found adequate support for a brief three-factor version of the EDE-Q in vegans, however this model does not meet the minimum recommendations for factor analysis by containing just two items per latent variable [23]. Taken together, it is clear that exploring the factor structure of common eating disorder tools in these groups remains a critical future research avenue. These preliminary findings also potentially suggest that the theoretical constructs that eating disorder tools are designed to assess may not necessarily be suitable to capture the unique eating attitudes and behaviours of vegetarians and vegans. While it is the case that these tools were originally developed in omnivorous groups prior to the wide uptake of meat-free diets, their generalisability to vegetarian and vegan samples is often assumed [19]. Indeed, potentially confusing eating disorder measure items have been previously noted (see Table 1 in McLean et al. [11]), confirming concerns around the face validity of such measures.

**Table 1:** Participant demographic characteristics for qualitative interview phases (Phase 1 and Phase 2)

	Phase 1	Phase 2
*N* (% (*n*))	25	18
Vegetarian	16.0 (4)	16.7 (3)
Vegan	20.0 (5)	16.7 (3)
Lived experience*	32.0 (8)	27.8 (5)
Psychologist	16.0 (4)	16.7 (3)
Dietician	16.0 (4)	22.2 (4)
Age (*M* (*SD*))	37.1 (13.1)	36.1 (11.6)
Vegetarian	35.8 (18.6)	28.0 (3.6)
Vegan	38.6 (12.7)	47.7 (13.9)
Lived experience	38.25 (14.7)	36.0 (10.8)
Psychologist	40.4 (11.4)	33.3 (1.5)
Dietician	29.8 (5.5)	38.0 (16.3)
Gender (% female (*n*))	80.0 (20)	77.8 (14)
Vegetarian	75.0 (3)	33.3 (1)
Vegan	40.0 (3)	33.3 (1)
Lived experience	100.0 (8)	100.0 (5)
Psychologist	100.0 (4)	100.0 (3)
Dietician	75.0 (4)	100.0 (4)

*Lived experience group consisted of five vegetarian/vegan and three omnivore participants in Phase 1 and two vegetarian/vegan and three omnivore participants in Phase 2

There are several specialised tools to assess eating disorder symptoms in at-risk groups such as athletes and people with diabetes (e.g., [24, 25]). For example, the Diabetes Eating Problem Survey (DEPS-R) is a revised 16-item self-report tool developed to screen for disordered eating in people with type 1 diabetes. However, there is currently no available tool to uniquely target people who follow a vegetarian or vegan diet. Current measures may be inappropriate for use in vegetarians and vegans for several reasons. First, vegetarianism and veganism require a degree of dietary restraint to ensure meat and/or animal products are not consumed [11]. Current measures may not be able to decipher the fundamental driving factors behind dietary restraint versus cognitive restraint in these groups (e.g., to follow a vegetarian or vegan diet versus to influence weight or shape), resulting in overall higher levels of disordered eating measured. Second, current measures appear not to be able to identify eating behaviours that are unique to individuals following a vegetarian or vegan diet, such as displaying self-control around food in public or extensively reading ingredient lists of foods to ensure meat and/or animal products are not consumed [11]. For these reasons, it is potentially important to employ a screening tool designed specifically for individuals following a vegetarian or vegan diet when assessing eating disorder symptomology in these groups. A valid self-report tool designed for this purpose could allow clinicians and researchers a quick, inexpensive, and efficient way to potentially identify individuals who may need further evaluation or intervention. Therefore, the present study aimed to develop and preliminary validate a novel screening tool to identify eating disorder symptoms in individuals following a vegetarian and vegan diet.

## Phase 1: Conceptual framework development

This stage of development aimed to create a conceptual framework for the presentation of eating disorder symptoms in vegetarians and vegans.

### Methods

#### Transparency and openness

Methods for the following study phases were approved by Monash University Human Research Ethics Committee (Project ID: 30651).

#### Participants

A total of 25 participants, including vegetarians (*n* = 4) and vegans (*n* = 5) without lived eating disorder experience; vegetarians, vegans, and omnivores with lived eating disorder experience (*n* = 8); and psychologists (*n* = 4), and dieticians (*n* = 4) took part in either a semi-structured interview or focus group. Participants were recruited through various social media advertisements (e.g., private vegan Facebook groups and community noticeboards), established participant recruitment databases, and researchers’ professional networks (e.g., local and national eating disorder charities, colleagues within the eating disorder field). Participants were required to be 18 years or over, residing in Australia, and have access to video-conferencing equipment. Those taking part as a community-based participant (i.e., vegetarians, vegans, omnivores, lived experience) were required to adhere to a vegetarian or vegan diet and/or have lived eating disorder experience, whereas those taking part as a psychologist or dietician were required to have self-reported professional experience within the eating disorder field. Taking into consideration sample variability, participant availability, age, gender, dietary adherence, and if applicable, lived eating disorder history or experience in the field, participants were invited to take part in the study between March and April of 2022. Sample size was determined by data saturation whereby no new information emerged and no new themes could be generated within and between participant groups from the interviews [26].

#### Data collection

First, participants were required to complete a short expression of interest survey detailing basic demographic characteristic information, including age, gender, dietary adherence, and eating disorder history if applicable. If inclusion criteria were met, community-based participants were invited to an individual semi-structured interview with two researchers hosted via video conferencing platform, Zoom. Psychologist and dietician participants were invited to share their professional opinion on vegetarian and vegan eating attitudes and behaviours through a combination of focus groups and one-on-one interviews via Zoom. Each session lasted 30–60 min (*M* = 41.20, *SD* = 6.40) and were audio recorded.

The interview script was targeted towards understanding the eating habits of vegetarians and vegans, the relationship between vegetarianism, veganism, and disordered eating, and the applicability of eating disorder instruments in vegetarian and vegan groups. The psychologist and dietician interview script also focused on the diagnosis, treatment, and recovery of eating disorders in individuals following a vegetarian and vegan diet.

#### Data analysis

Interviews were first de-identified and transcribed by a member of the research team. Each transcript was then imported into an individual excel document with text grouped under each interview question, whereby data were analysed using an inductive approach. Three researchers were involved in data analysis, employing the following steps: transcripts were read for familiarity, relevant quotations were highlighted, codes were developed, and codes were merged into themes and subthemes. At the completion of data analysis, the research team discussed codes and themes generated, whereby those with discrepancies were deleted, added, or collapsed until consensus was reached [26].

### Results

Participants in Phase 1 comprised primarily women (80.0%) and had an average age of 37.1 years (*SD* = 13.1; see Table 1).

**Fig. 1:**
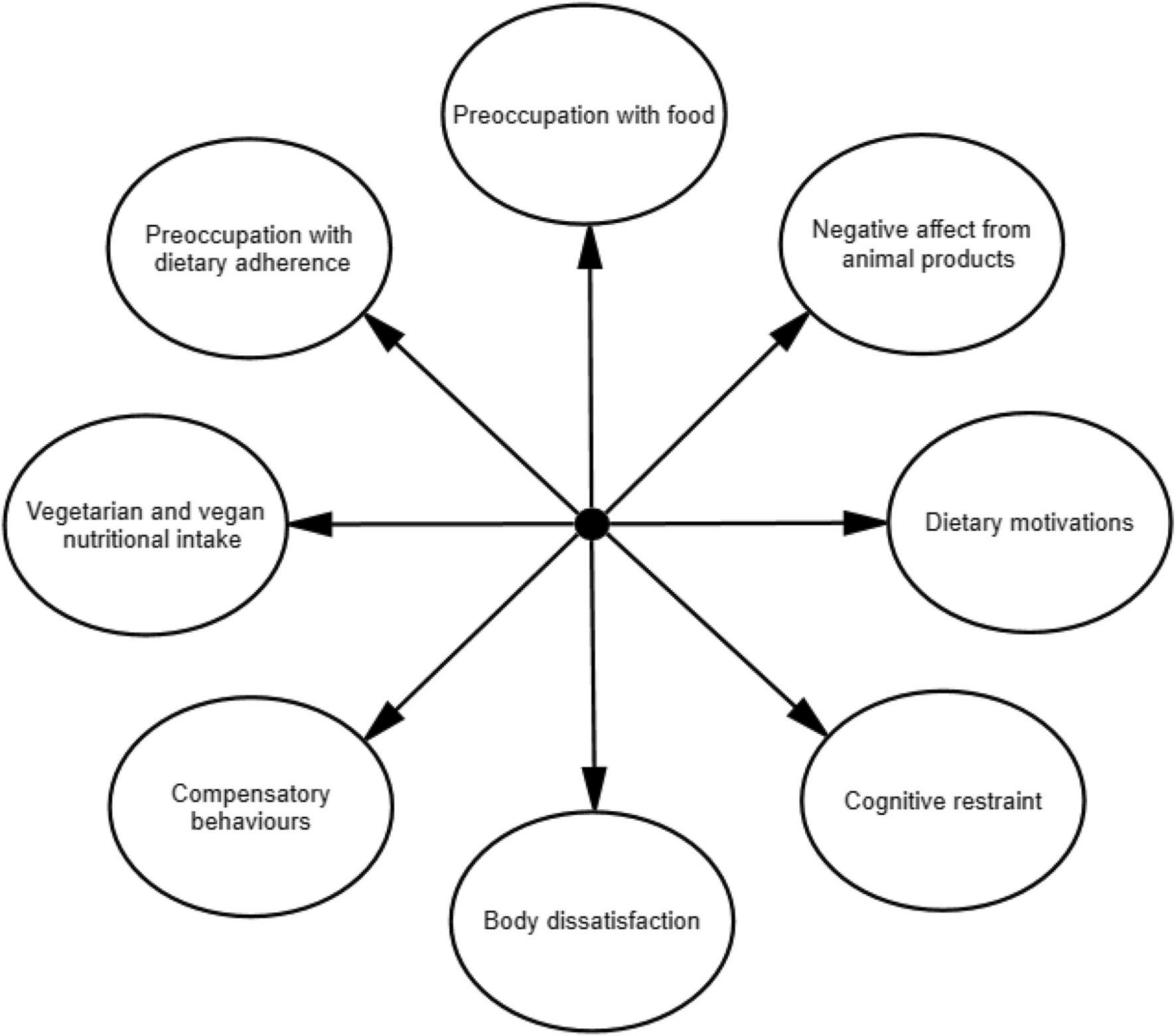
V-EDS conceptual framework

Based on the interview data generated, eight key themes formed our conceptual framework for the presentation of eating disorder symptoms in vegetarians and vegans. As per Fig. 1, we note the inclusion of vegetarian and vegan-specific themes (e.g., dietary motivations), in addition to several core eating disorder themes (e.g., body dissatisfaction; see Additional File 1 for a description of themes).

## Phase 2: Item generation and pretesting

This phase involved developing an exhaustive list of prospective items using the conceptual framework developed in Phase 1 and piloting them for discussion and feedback.

### Methods

#### Participants

A total of 18 participants, including vegetarians, vegans, and omnivores with and without lived eating disorder experience, psychologists, and dieticians took part in semi-structured interviews. Participants were recruited using social media, snowballing techniques, and participant recruitment databases and were required to adhere to the same eligibility criteria as per Phase 1. Based on demographic variability, participants were invited to take part in the study via Zoom between July and August of 2022. A total of 38.9% of participants from Phase 1 also took part in this phase of the study, whereby sample size was determined by data saturation if no new information emerged and no new themes could be generated within and between participant groups [26].

#### Data collection

Data collection methods were identical to Phase 1 with each session lasting 30–60 min (*M* = 52.50, *SD* = 14.63). Cognitive debriefing techniques were integrated to examine the degree to which the developed items resonated with relevant personal or professional experiences, that the items were understandable, to rule out any unclear or ambiguous items, and  to ensure readability of the items.

#### Data analysis

Interviews were first de-identified manually by a member of the research team, then transcribed using the confidential third-party platform, Otter.ai [27]. Additional data analysis processes followed that of Phase 1, with conclusions reached for each individual item by examining within and between subgroup data.

### Results

Participants in Phase 2 comprised primarily women (80.0%) and had an average age of 36.1 years (*SD* = 11.6; see Table 1). The process of item generation led to the creation of an exhaustive list of 163 items across the eight conceptual framework themes. We further examined current and widely available eating disorder screening tools, including the EDE-Q [20], SCOFF [28], Eating Pathology Symptoms Inventory (EPSI; [29]), and Eating Disorder Inventory (EDI; [30]), to confirm that key item themes had been integrated. However, this did not result in the addition of any new items. Finally, psychologists and dieticians were invited to nominate items that they considered to be missing from the list or required further clarification. Based on the interview data generated, pilot testing of the items resulted in a reduced, revised questionnaire of 53 items.

## Phase 3: Testing and scale construction

This phase of development aimed to test the preliminary set of developed items on a large sample of participants to aid in item reduction and final scale formation.

### Methods

#### Participants

Through social media advertisements and participant recruitment databases, a total of 961 participants were recruited to take part in an online survey. Inclusion criteria required that participants were 18 years or over and residing in Australia to be eligible. Dietary status was assessed using a two-tier classification previously described in McLean et al. [6]. Due to our specific focus on vegetarian and vegan eating behaviours, meat-reducers (i.e., flexitarians, pescatarians, semi-vegetarians) were excluded from the final dataset and all analyses (*n* = 80). This resulted in a sample of 881 participants, comprising 230 vegetarians and 651 vegans. As there were a great deal more vegan participants than vegetarians, a random subsample of 230 vegan participants were selected to make the sample size of each dietary group equitable [31]. This approach was taken to ensure the developed screening tool would be applicable to both groups, resulting in a final sample of 460 participants.

#### Measures

Demographic characteristic information including age, gender, ethnicity, religion, and highest completed education were collected. Participants who stated they followed a vegetarian or vegan diet were asked additional specific questions around their dietary adherence, including length of dietary adherence and dietary motivations, with possible categorical responses being animal welfare, family tradition, financial, health, spiritual beliefs, environment, weight control, food sensitivity or intolerance, and taste, texture, and /or smell preferences.

Participants responded to the preliminary 53-item self-report Vegetarian Vegan Eating Disorder Screener (V-EDS) designed to assess the unique eating disorder symptomology of vegetarians and vegans over the past seven days. The reference time of seven days was chosen to provide optimal recall accuracy and allow for the V-EDS to track changes of symptoms over a short timeframe. The preliminary V-EDS consisted of 11 dietary characteristic items designed to provide important information about the respondent’s dietary attitudes (e.g., *“The thought of accidentally eating meat causes you significant distress”*) along a 5-point Likert scale from s*trongly disagree* to *strongly agree.* While these items do not inherently indicate eating pathology when considered in isolation, we discovered through Phase 1 and 2 interviews that these attitudes are important to the respondent’s history. The questionnaire is followed by 42 behavioural and attitudinal items (e.g., *“Has the way you think about food become intrusive?”*) designed to measure the presence of eating disorder pathology, rated along a 5-point Likert scale from *no days* to *every day*, with higher scores indicating higher eating disorder pathology.

#### Procedure

Participants were advertised with a link to the online survey titled “*Development of a New Eating Disorder Screener for Vegetarians and Vegans*” and presented with the Explanatory Statement to provide informed consent. To ensure high data quality, participants were directed to complete a Captcha, a simple arithmetic question presented as an image, and two attention checks presented throughout the survey [32]. If participants failed any of the above quality or attention checks, they were excluded from the online survey and their IP address was not allowed to re-enter the survey. Participants then responded to demographic characteristic information, their weight and height to calculate BMI (weight in kg/height in m^2^), and the V-EDS. Finally, participants noted if they had ever received an eating disorder or mental health diagnosis from a health professional. Participants were given the opportunity to enter the draw to win one of four $AU50 gift cards (~ $US35).

#### Statistical analysis

SPSS Version 27.0 [33] was used to conduct frequencies and descriptive statistics for participant demographic characteristics. Stata 17 software [34] was used to conduct Item Response Theory (IRT) analysis whereby the 42 scored V-EDS items were examined using two-parameter logistic (2PL) models in combined and separate vegetarian and vegan samples to estimate individual parameters for difficulty and discrimination. Items were deemed unacceptable if they had limited ability to discriminate between low- and high-ability participants or discrimination coefficients with *p* > 0.05. The 11 dietary characteristic items were not included in the IRT analysis as they are not designed to be scored along a measurement scale, but rather used to provide categorical insight into a respondent’s eating attitudes and behaviours when considered alongside the V-EDS scored items. The dietary characteristic items were assessed for final inclusion between the authorship group.

### Results

In the present study, the most common primary motivation for adhering to a vegetarian diet was animal welfare (53.9%), the environment (19.6%), and taste, texture, and/or smell preferences (10.0%), with a median diet length of 10.0 years (*IQR* = 17.0). In the vegan sample, the most common primary dietary motivation was animal welfare (75.7%), health (8.3%), and the environment (7.8%), with a median diet length of 7.0 years (*IQR* = 7.0). Table 2 presents descriptive statistics for the overall sample and subgroups, and test statistic results.

**Table 2:** Phase 3 participant demographic characteristics

		Total (*n* = 460)	Vegetarian (*n* = 230)	Vegan (*n* = 230)	Statistics
Age	*M (SD)*	32.6 (10.4)	32.4 (10.4)	32.9 (10.5)	*t*(458) = −0.49, *p* = .622
Gender	% female (*n*)	83.9 (386)	81.7 (188)	86.1 (198)	*χ*^*2*^(3) = 2.54, *p* = .469
BMI	*M (SD)*	24.7 (5.7)	25.5 (6.2)	23.9 (5.0)	*t*(458) = 3.13, *p* = .002*
Ethnicity	% Australian (*n*)	51.3 (236)	53.9 (124)	48.7 (112)	*χ*^*2*^(7) = 11.64, *p* = .113
Religion	% no religion (*n)*	78.9 (363)	77.4 (178)	80.4 (185)	*χ*^*2*^(9) = 22.59, *p* = .007*
Education	% ≥ Bachelors (*n*)	65.4 (301)	68.3 (157)	62.6 (144)	*H*(1) = 2.30, *p* = .130
Eating disorder diagnosis	% (*n*)	25.6 (118)	27.4 (63)	23.9 (55)	*χ*^*2*^(2) = 0.75, *p* = .688
Mental health diagnosis	% (*n*)	45.2 (208)	43.0 (99)	47.4 (109)	*χ*^*2*^(1) = 0.88, *p* = .349

*p = .01. Independent samples *t*-test or Mann–Whitney U test with main effect of dietary group was used to evaluate continuous and ordinal dependent variables; Chi-square test of independence for categorical row variables. Gender = male, female, non-binary, prefer not to disclose, prefer to self-describe; Ethnicity = English, Irish, Scottish, Italian, German, Chinese, Australian, other; Religion = no religion, Catholic, Anglican, Uniting Church, Presbyterian, Buddhism, Islam, Greek Orthodox, Baptist, Hinduism, other; Education = Doctoral degree, Master’s degree, Bachelor’s degree with Honours/Graduate Diploma/Graduate Certificate, Bachelor’s degree, Diploma/Advanced Diploma, Trade or Certificate III/IV, Certificate I/II, Year 12/Secondary School Certificate, Year 11 or below, other; Eating disorder diagnosis levels = yes current diagnosis, yes previous diagnosis, no; Mental health diagnosis = yes, no

In separate 2PL models of vegetarians and vegans, parameter estimates indicated that a number of items were not well-suited to measure the latent variable with poor discrimination and difficulty parameters. Such findings were compared between dietary groups, with items with the poorest parameter estimates iteratively removed until well-performing items were remaining. Next, items with similar wording (e.g., “*Have you restricted large amounts of food to change the way your body looks?*”, “*Have you excluded large amounts of food to change the way your body looks?*”) were compared between vegetarian, vegan, and combined 2PL models with poorer functioning items removed. This resulted in a reduced, revised questionnaire of 12 scored items. The final IRT of the reduced 12-item scale in a combined and separate sample of vegetarians and vegans showed the test information function was single-peaked and reached its maximum in the middle-left of the distribution of the latent variable. Several dietary characteristic items were also removed until a consensus of six items was reached between the authorship group. The removal of items was based on those which had similar wording or addressed a similar expected outcome.

## Phase 4: Psychometric assessment

This phase of development aimed to conduct psychometric analysis of the final version of the developed scale to validate its use in a non-clinical community sample of vegetarians and vegans. In doing so, we elected to undertake exploratory factor analysis to discover the underlying factor structure of the V-EDS in separate groups of vegetarians and vegans. It is expected that the V-EDS will support good to excellent initial psychometric properties.

### Methods

#### Participants

A total of 1095 participants were recruited through social media advertisements and participant recruitment databases to take part via an online survey. Participants were required to adhere to identical inclusion criteria as per Phase 3, with meat-reducers and omnivores excluded from all analyses (*n* = 445). This resulted in a final sample of 650 participants (vegan = 405, vegetarian = 245). A smaller subtest of the sample (*n* = 71, 10.9%) took part in a 14-day test–retest reliability study comprising a further 22 vegetarian and 49 vegan participants.

#### Measures

Similar to Phase 3, participants responded to demographic characteristic information and dietary adherence questions.

The final Vegetarian Vegan Eating Disorder Screener (V-EDS) is an 18-item self-report screening tool designed to assess the unique eating disorder symptomology of vegetarians and vegans over the past seven days (see Additional file 2). The V-EDS consists of 6 dietary characteristic items designed to provide important background information to the respondent’s dietary attitudes, rated along a 5-point Likert scale from s*trongly disagree* to *strongly agree.* The dietary characteristic items are followed by 12 behavioural and attitudinal items (e.g., *“Has the way you think about food become intrusive?”*) designed to measure the presence of eating disorder pathology, rated along a 5-point Likert scale from *no days* to *every day*. Higher scores on the V-EDS indicated greater eating disorder pathology.

The Eating Disorder Examination-Questionnaire (EDE-Q; [20]) is a self-report tool designed to measure the attitudinal and behavioural symptoms of eating disorders over the past 28 days. The EDE-Q comprises 28 items, including 22 attitudinal items and six open-response behavioural frequency items, whereby participants respond to each attitudinal item (e.g., *“Have you had a definite fear of losing control over eating?”*) along a 7-point Likert scale ranging from *not at all* to *markedly*. A global score is calculated by summing the four subscales: Eating Concern, Weight Concern, Shape Concern, and Restraint, and dividing by four (i.e., the number of subscales), with higher scores indicating greater eating disorder pathology. The EDE-Q has demonstrated good internal consistency, with a score of 0.96 in the total sample for the present study. As the EDE-Q is the most widely recognised clinical tool and forms part of the eligibility criteria for Medicare-subsidised treatment in Australia, it was included in the present study to assess the convergent validity of the V-EDS.

The Depression, Anxiety, and Stress Scale (DASS-21; [35]) is a 21-item self-report scale designed to assess the negative emotions associated with depression, anxiety, and stress over the past seven days. Each negative emotional subscale contains seven items and are responded to along a 4-point Likert scale from *never* to *almost always*. A subscale score is calculated by summing the seven associated items and multiplying by two, with higher scores indicating greater negative emotions. The DASS-21 has demonstrated good internal consistency, with a score of 0.93, 0.84, and 0.89 for the Depression, Anxiety, and Stress subscales, respectively, in the total sample for the present study. As depression traits are shown to have moderate divergent patterns with eating disorders [36], the DASS-21 was included in the present study to examine the divergent validity of the V-EDS.

#### Procedure

As with Phase 3, participants were advertised with a link to the online survey and presented with the Explanatory Statement to provide informed consent. Participants then responded to demographic characteristic information, their weight and height to calculate BMI, and the V-EDS, EDE-Q, and DASS-21 in randomised order. Finally, participants noted if they had ever received an eating disorder or mental health diagnosis from a health professional and were given an opportunity to enter a gift card draw. Identical data quality processes were integrated as per Phase 3.

#### Statistical analysis

SPSS Version 27.0 [33] was used to conduct frequencies and descriptive statistics for participant demographic characteristics, reliabilities, validities, and exploratory factor analysis (EFA). The vegetarian and vegan samples each had one variable with missing data at random and was imputed with the variable median [37]. CFA was conducted separately for vegetarians and vegans to confirm fit of the V-EDS model discovered via Phase C. Diagonally weighted least squares was used as the estimation method as it provides a robust estimation when dealing with ordinal data that violates the assumption of multivariate normality [38]. Adequacy of model fit was evaluated by examining several fit indices as judged using a two-index presentation strategy [39]. Relative model fit was judged with a specific focus on Comparative Fit Index (CFI), with ≥ 0.90 being acceptable and ≥ 0.95 being excellent [39]. Absolute model fit was judged with a focus on point estimate of root mean square error of approximation (RMSEA), with < 0.05 demonstrating good fit, between 0.05 and 0.08 acceptable fit, and > 0.08 poor fit [40]. Information linked to other fit indices were also reported and considered, including *x*^2^ value, Tucker-Lewis Index (TLI), and standardised root mean square residual (SRMR; [41–43]).

Internal consistency of the 12 scored V-EDS items were calculated using Cronbach’s coefficient alpha (*α*) for each dietary group. Convergent and discriminant validity were calculated using Pearson correlations (*r*) between the V-ED scored items and scores on the EDE-Q and DASS-21 subscales, respectively, for each group. Due to an insufficient sample size required to obtain 95% confidence in the test–retest reliability arm [44], analyses were not conducted for this statistic.

### Results

#### Descriptive statistics

In the present study, the most common primary motivation for adhering to a vegetarian diet was animal welfare (58.8%), the environment (16.3%), and taste, texture, and/or smell preferences (9.0%), with a median diet length of 10.0 years (*IQR* = 16.0). In the vegan sample, the most common primary motivation was animal welfare (79.8%), health (8.9%), and the environment (7.4%), with a median diet length of 8.0 years *IQR* = 8.0). Table 3 presents descriptive statistics for the overall sample and subgroups, and omnibus test results to compare the vegetarian and vegan groups.

**Table 3:** Phase 4 participant demographic characteristics

		Total (*n* = 650)	Vegetarian (*n* = 245)	Vegan (*n* = 405)	Statistics
Age	*M (SD)*	34.3 (11.0)	34.2 (10.9)	34.4 (11.2)	*t*(648) = −0.18, *p* = .855
Gender	% female (*n*)	84.8 (551)	86.1 (211)	84.0 (340)	*χ*^*2*^(4) = 0.92, *p* = .922
BMI	*M (SD)*	24.5 (5.3)	25.2 (6.1)	24.1 (11.2)	*t*(647) = 2.55, *p* = .011*
Ethnicity	% Australian (*n*)	45.1 (293)	41.6 (102)	47.2 (191)	*χ*^*2*^(7) = 13.15, *p* = .068
Religion	% no religion (*n)*	82.6 (537)	79.2 (194)	84.7 (343)	*χ*^*2*^(10) = 16.89, *p* = .077
Education	% ≥ Bachelors (*n*)	68.9 (448)	73.9 (181)	65.9 (267)	*H*(1) = 7.37, *p* = .007**
Eating disorder diagnosis	% (*n*)	13.6 (139)	12.7 (55)	14.1 (84)	*χ*^*2*^(2) = 2.16, *p* = .34
Mental health diagnosis	% (*n*)	40.8 (265)	40.4 (99)	41.0 (166)	*χ*^*2*^(1) = 0.02, *p* = .884
V-EDS Global	*Med (IQR)*	4.0 (13.0)	5.0 (16.0)	4.0 (11.0)	*H*(1) = 5.06, *p* = .025*

**p < .01, *p < .05. Independent samples *t*-test or Mann–Whitney *U* test with main effect of dietary group was used to evaluate continuous and ordinal dependent variables; Chi-square test of independence for categorical row variables. Gender = male, female, non-binary, prefer not to disclose, prefer to self-describe; Ethnicity = English, Irish, Scottish, Italian, German, Chinese, Australian, other; Religion = no religion, Catholic, Anglican, Uniting Church, Presbyterian, Buddhism, Islam, Greek Orthodox, Baptist, Hinduism, other; Education = Doctoral degree, Master’s degree, Bachelor’s degree with Honours/Graduate Diploma/Graduate Certificate, Bachelor’s degree, Diploma/Advanced Diploma, Trade or Certificate III/IV, Certificate I/II, Year 12/Secondary School Certificate, Year 11 or below, other; Eating disorder diagnosis levels = yes current diagnosis, yes previous diagnosis, no; Mental health diagnosis = yes, no

#### Dietary characteristic items

##### Item 1: I’m motivated to eat my food choices for…?

**Fig. 2:**
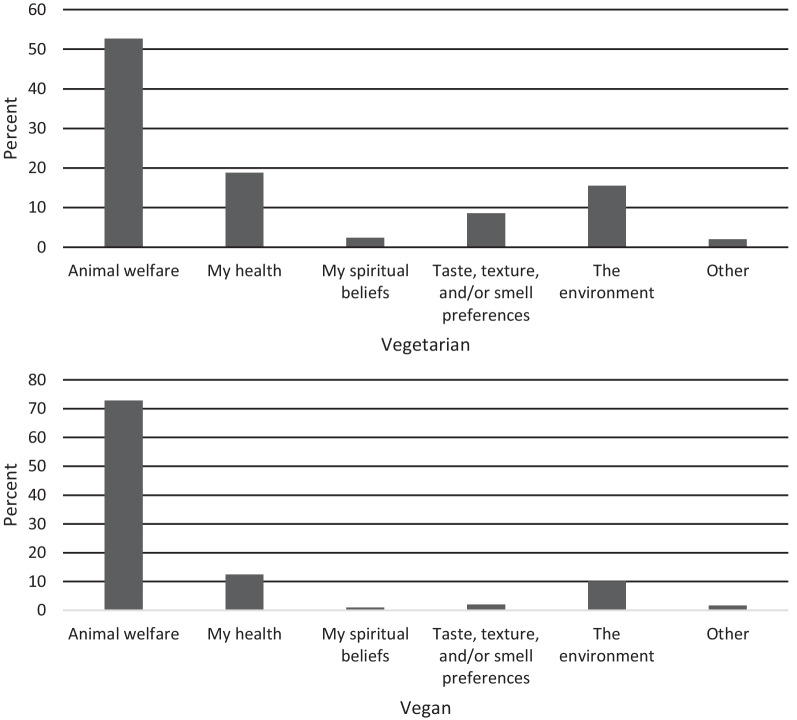
Item 1 response characteristics across dietary groups

This item aimed to understand the primary dietary motivation driving the respondent’s adherence to a vegetarian or vegan diet (Fig. 2). Respondents are presented with five categorical options shown to be the most common dietary motivations (e.g., [6]).

##### Item 2: Your vegetarian/vegan diet is part of your identity

**Fig. 3:**
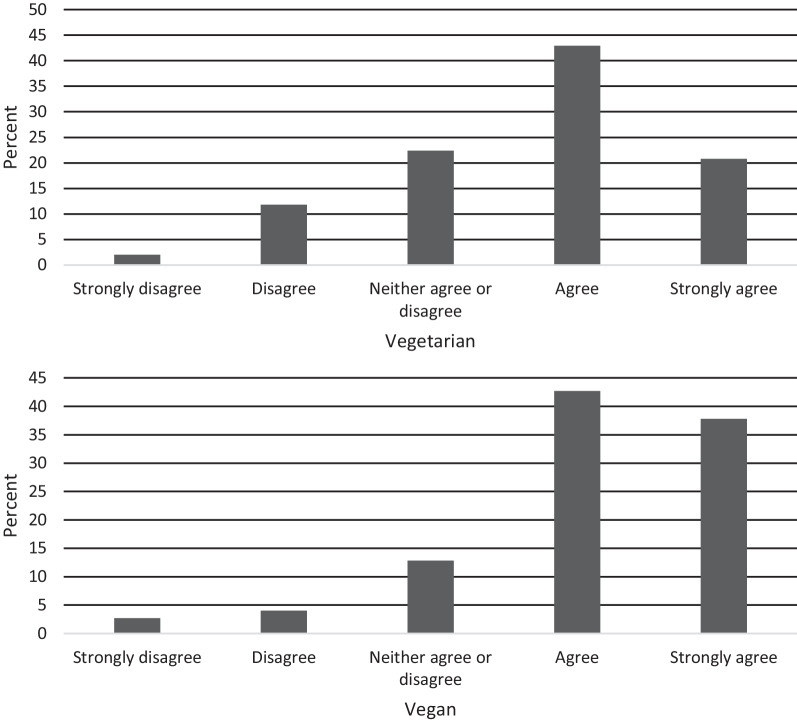
Item 2 response characteristics across dietary groups

Item 2 aimed to examine the degree the respondent perceives their dietary adherence to be tied to their identity (Fig. 3). We found significant differences in scores between dietary groups (*t*(648) = -5.14, *p* < 0.001, *d* = 0.42), with vegans more likely to strongly agree with this item.

##### Item 3: A balanced diet can include eating processed plant-based products (e.g., mock meats)

**Fig. 4:**
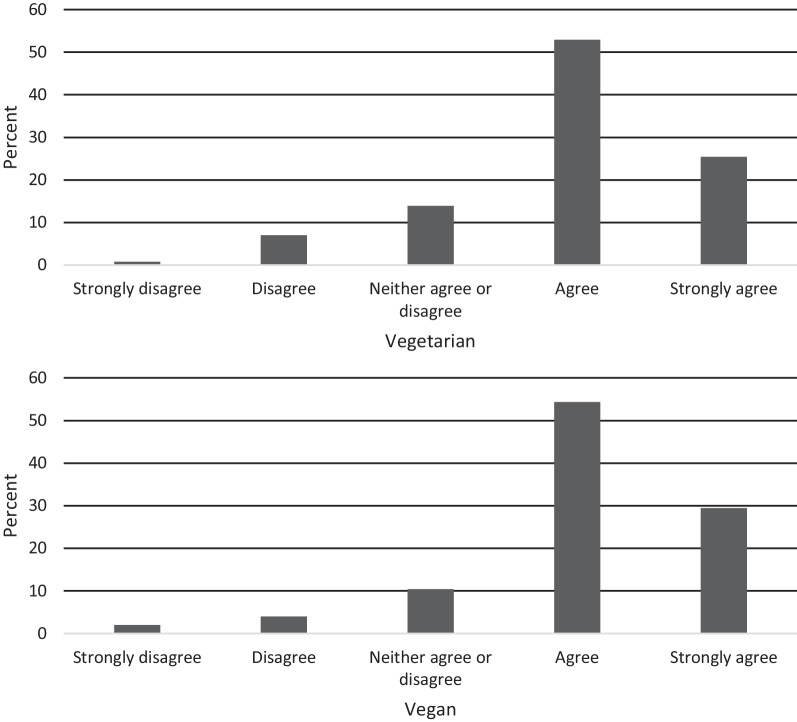
Item 3 response characteristics across dietary groups

This item aimed to assess the respondent’s rigidity around the consumption of processed plant-based products which may provide insight into “clean eating” practices (i.e., a diet approach of eating primarily unprocessed and unrefined foods; [45]) often associated with vegetarian and vegan diets (Fig. 4). We found no significant differences in scores between dietary groups (*t*(648) = −1.45, *p* = 0.147, *d* = 0.12.

##### Item 4: You are willing to introduce meat to your diet if it is vital for your survival

**Fig. 5:**
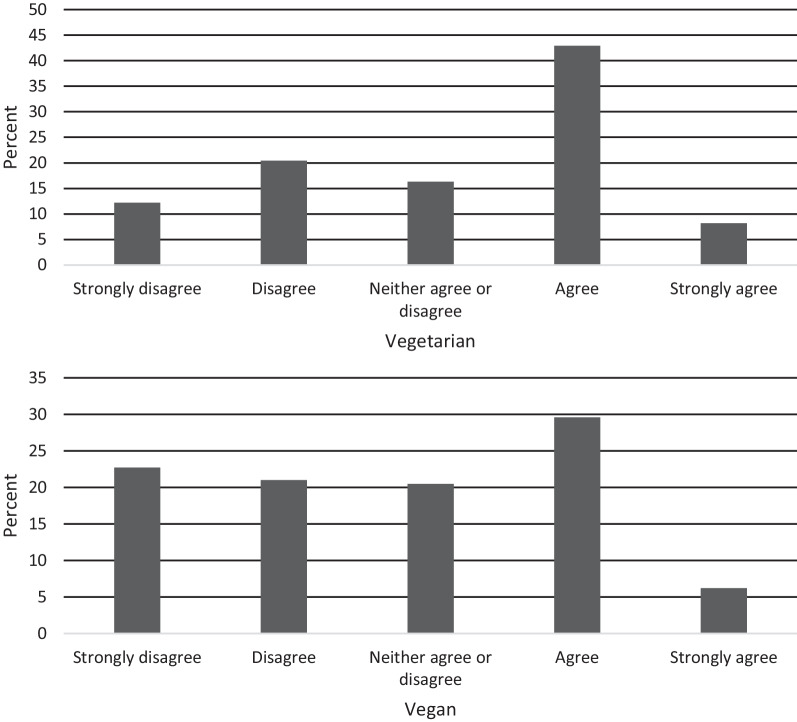
Item 4 response characteristics across dietary groups

Item 4 was developed to explore the respondent’s openness to the introduction of meat if necessary for their physical and/or mental health as part of eating disorder recovery. If accompanied by higher V-EDS scores and subsequent diagnosis of an eating disorder, this item may provide clinicians with potential insights into the respondent’s receptiveness to the consumption of meat and can be used to approach future client discussions on the topic. We found significant differences in scores between dietary groups (*t*(648) = 3.85, *p* < 0.001, *d* = 0.31), with vegans demonstrating stronger disagreement with the sentiment (Fig. 5).

##### Item 5: The thought of accidentally eating meat causes you significant distress

**Fig. 6:**
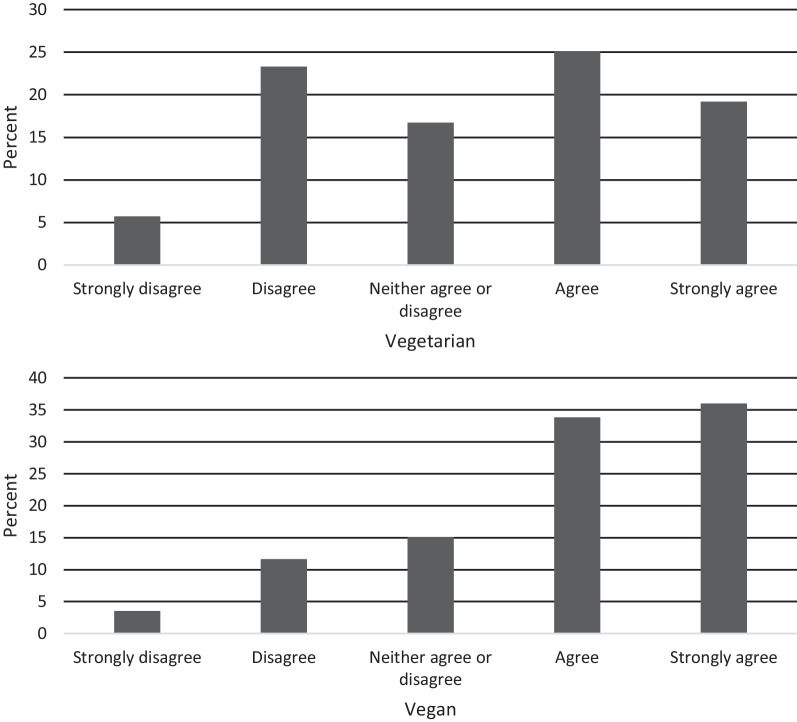
Item 5 response characteristics across dietary groups

Item 5 explores a respondent’s potential aversion to the consumption of meat (Fig. 6). There were statistically significant differences in scores between dietary groups (*t*(648) = −5.20, *p* < 0.001, *d* = 0.42), with vegans demonstrating overall stronger agreement with the sentiment.

##### Item 6: Removing meat and/or animal products from your diet allows you to control the way your body looks

**Fig. 7:**
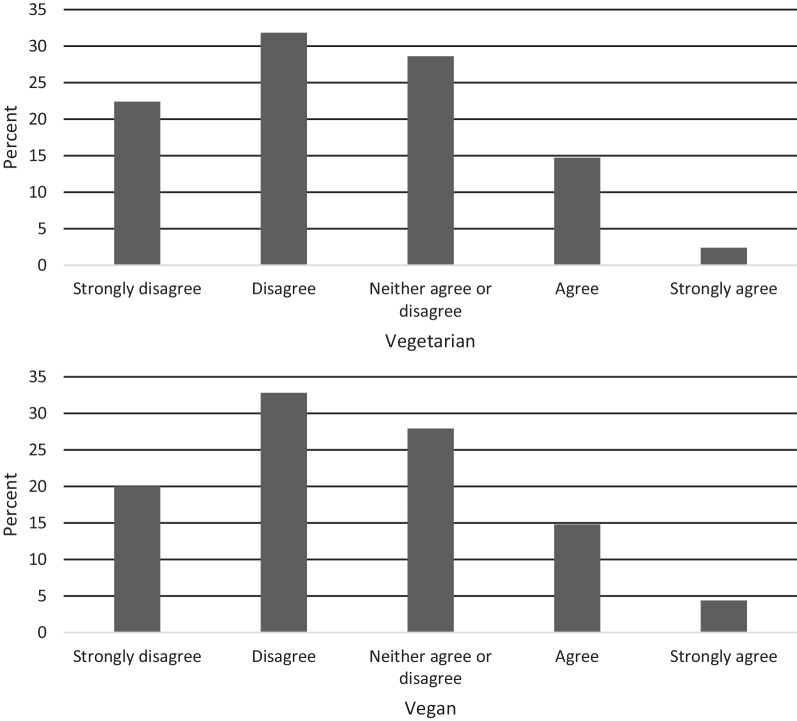
Item 6 response characteristics across dietary groups

This item was developed to pick up on whether a respondent may be engaging in a vegetarian or vegan diet for body modification reasons, and thus may be at a higher risk for eating disorder development (Fig. 7). There were no significant differences in scores between dietary groups (*t*(648) = −0.91, *p* = 0.364, *d* = 0.07).

#### Confirmatory factor analysis

Model fit of the one-factor model was judged to be acceptable in separate analysis of vegetarians and vegans. See Table 4 for individual model fit indices across dietary groups.

**Table 4:** Confirmatory factor analysis fit statistics of the V-EDS by dietary adherence

	*x*^2^ (*df*)	CFI	TFI	RMSEA [95% *CI*]	SRMR
Vegetarian	143.38 (54)*	.99	.99	.06 [0.7, .10]	.05
Vegan	147.96 (54)*	.99	.99	.07 [.05, .08]	.04

CFI = comparative fit index, TFI = Tucker Lewis index, RMSEA = root mean square error of approximation, SRMR = standardised root mean square residual. *p < .001. Vegetarian *n* = 245, vegan *n* = 405

#### Internal consistency and construct validity

Table 5 summarises the internal consistency reliability, convergent bivariate Pearson correlations with the EDE-Q global score, and discriminant bivariate Pearson correlations with the DASS-21 subscales across dietary groups. Internal consistency, as measured by Cronbach’s alpha, was excellent in separate vegan and vegetarian groups. Convergent correlations were very strong in strength, demonstrating excellent convergent validity of the V-EDS with the EDE-Q. Divergent correlations with the DASS-21 depression subscale was moderate in strength, demonstrating moderate discriminant validity.

**Table 5:** Psychometric characteristics of the V-EDS by dietary adherence

		α	EDE-Q	DASS-21		
				1	2	3
Vegetarian		0.96	.88**	.45**	.42**	.41**
Vegan		0.95	.87**	.55**	.56**	.55**

**p < .001. 1 = Depression subscale, 2 = Anxiety subscale, 3 = Stress subscale. Vegetarian *n* = 245, vegan *n* = 405

## Discussion

Eating disorders remain one of the most underdiagnosed mental illnesses, with early detection and intervention known to significantly improve the speed of recovery and symptom severity [46, 47]. Furthermore, some “normal” vegetarian and vegan eating attitudes and behaviours may overlap with eating disorder symptomology, and therefore such practices may be inappropriately captured by existing eating disorder instruments [11]. To address this gap, the present study employed a rigorous methodological design aimed at developing and preliminary validating a novel eating disorder screening tool for individuals following a vegetarian and vegan diet, the Vegetarian Vegan Eating Disorder Screener (V-EDS). The V-EDS was found to support a unidimensional model comprising six dietary characteristic items and 12 core behavioural and attitudinal items and there appear to be strong psychometric properties for use in non-clinical, community samples of vegetarians and vegans.

The V-EDS development relied on the personal and professional expertise of a wide range of participants across four phases of construct and item development. The V-EDS is a relatively brief, 18-item, self-administered screening tool designed to potentially identify eating disorder risk across clinical or research settings in individuals following a vegetarian or vegan diet. Demonstrating excellent psychometric properties, each item of the V-EDS was deemed to measure the same underlying dimension as supported by CFA. The V-EDS also correlated strongly with the widely employed diagnostic tool, the EDE-Q, and therefore may possibly, with further validation, be considered a candidate as a standalone measure or when combined with the EDE-Q for comprehensive assessment. The inclusion of the dietary characteristic items offers a novel distinguishing feature which can be used to assist healthcare professionals to understand how the patient’s dietary attitudes may integrate with their eating disorder symptomology. Developed from themes generated during in-depth interviews with eating disorder clinicians, these items can be used as “conversation starters” to decipher a patient’s dietary adherence and attitudes. For example, if accompanied by higher V-EDS scores and subsequent diagnosis of an eating disorder, Item 4 (“*You are willing to introduce meat to your diet if it is vital for your survival*”) may provide clinicians with potential insight into their patient’s dietary flexibility which may be required to achieve recovery. Indeed, the importance of understanding dietary motivations and dietary flexibility within the context of a vegetarian and vegan diet emerged as important themes in this study and may provide insight into a patient’s eating disorder practices [48, 49]. For example, if a patient is motivated to adhere to a vegetarian or vegan diet for animal welfare or ecological concerns, do they incorporate other ethical lifestyle practices, such as eliminating products tested on animals or prioritising sustainability. It could also be useful to explore whether the client places additional dietary restrictions within the context of their vegetarian or vegan diet. Ultimately the V-EDS could potentially fill a critical gap as a psychometrically-sound screening tool to detect and explore eating disorder risk among individuals who follow a vegetarian or vegan diet.

A screening instrument for the detection of eating disorder risk in vegetarians and vegans potentially offers a number of valuable opportunities. While dietary status may not necessarily be a risk factor for the development of an eating disorder, adherence to a vegetarian and vegan diet may provide an advantageous avenue to permit disordered eating behaviours in already vulnerable people [11]. The V-EDS can provide healthcare professionals with a potential starting point to begin to decipher their patient’s dietary adherence from their eating disorder symptomology. We encourage clinicians to explore their patient’s dietary adherence in a way that is sensitive, non-judgemental, and non-assumptive to allow for their beliefs to be respected but also challenged in a therapeutic manner. Next, the V-EDS can be used to encourage discussions related to vegetarian and vegan nutrition care to ensure the patient is meeting minimum dietary requirements. Last, the V-EDS may be used as a tool for specialised vegetarian and vegan eating disorder training. For example, the V-EDS could be included as part of professional development workshops for clinicians learning about the assessment and treatment of eating disorders in these growing populations.

### Strengths

This study developed, to our knowledge, the first eating disorder screening tool to specifically examine eating disorder risk in individuals who follow a vegetarian or vegan diet. We employed a rigorous methodological design that relied on critical input and review from a diverse participant group, including vegetarians, vegans, individuals with lived eating disorder experience, dieticians, and psychologists. Furthermore, each phase of this study recruited large sample sizes in accordance with minimum sample recommendations [50, 51]. Finally, the V-EDS may potentially overcome limitations of other commonly employed eating disorder tools to incorporate effective, quick, and inexpensive administration and scoring which provides reliable and valid results. While further research and validation are very much needed, the V-EDS may be a potential candidate for implementation in both healthcare and research settings [52].

### Limitations and future research

A limitation of this study is that we relied on the recruitment of convenience sampling through established participant recruitment databases, personal and professional networks, and social media groups. As a result, the generalisability of the findings, and therefore validation of the V-EDS, is limited to broader populations of vegetarians and vegans, including those who adhere to their diet for religious and/or cultural reasons. Next, a large portion of the sample in Phase 3 and 4 self-reported as having either a current or previous eating disorder diagnosis. This is likely a reflection of the targeted nature of our recruitment advertising via social media and therefore is subject to sampling bias. In addition, we categorised vegetarian and vegan participants according to Asher, Green [53] multistep classification. Ultimately, this process allowed for the collection of “clean” vegetarian and vegan samples [6], however does place value on dietary patterns over self-identity which may have influenced the interpretation of participant responses. Furthermore, this study is preliminary in nature, and therefore we were unable to conduct a full suite of psychometric properties of the V-EDS across all participant groups and demographics. We encourage future research to expand on the present study and examine the psychometric properties of the V-EDS in more diverse samples in terms of age, gender, ethnicity, geographic region, dietary motivations, and clinical background. Finally, we were unable to conduct test–retest reliability analysis due to insufficient sample size as a result of potential response bias. Future research should focus on assessing the consistency of the V-EDS across time points, but also administration methods (e.g., digital vs pen and paper) to determine whether the V-EDS can be used to track progress through eating disorder recovery.

## Conclusion

This study aimed to develop and preliminary validate a novel eating disorder screening tool for use in individuals following a vegetarian or vegan diet. We developed an 18-item tool, comprising six dietary characteristic items and 12 eating disorder scored items which demonstrated excellent initial psychometric properties. The V-EDS constitutes a promising instrument that could potentially be integrated as a standalone measure for initial screening in clinical and research settings, but also for more comprehensive assessment when combined with other gold-standard eating disorder tools, such as the EDE-Q and clinical interview. Ultimately, to our knowledge, the development of the V-EDS as the first eating disorder tool to capture unique vegetarian and vegan eating disorder symptomology offers an important step forward in understanding the complex relationship between vegetarianism, veganism, and eating disorders. Future research could build on our preliminary work and further validate the V-EDS across a wider range of diverse samples, including clinical backgrounds.

### Supplementary Information

**Additional file 1**. V-EDS conceptual framework themes.

**Additional file 2**. Vegetarian Vegan Eating Disorder Screener (V-EDS).

## Data Availability

The datasets generated and/or analysed from this study cannot be made available in line with ethics compliance.
