# Supplementary material for: Development and preliminary validation of a novel eating disorder screening tool for vegetarians and vegans: the V-EDS
Source: J Eat Disord. 2024 Jan 9;12:4. doi: 10.1186/s40337-024-00964-7 (PMC10775595; doi:10.1186/s40337-024-00964-7)
Supplement: Supplementary file 1 — Additional file 1. V-EDS conceptual framework themes. [file 40337_2024_964_MOESM1_ESM.docx]

**Additional File 1**

| Table A1. V-EDS conceptual framework themes | | |
| --- | --- | --- |
| Themes | Definition | Exemplar interview excerpts |
| Preoccupation with food | This theme is defined as excessive and constant thoughts about food which negatively affect ones self-worth (1). Food preoccupation is considered to be a core feature of eating disorder pathology (2). | *“There are different aspects of preoccupation with food. But because it's all centred around the calorie counting, for most people with an eating disorder, that is going to be the content of their thought pattern.”* - Psychologist |
| Negative affect from animal products | This theme refers to an aversion towards meat and/or animal products frequently experienced by people following to a vegetarian or vegan diet. The aversion may relate to the sight, smell, or taste of the meat and/or animal products and may be accompanied by feelings of disgust. Literature has demonstrated that feelings of meat disgust may predict stricter adherence to vegetarian and vegan diets (3). | *“The intake of animal products, like meat, have certain connotations to being repulsive or disgusting to intake into your body. It’s something that is almost like impure. I think that could also relate to motivations to go vegetarian or vegan.” -* Vegan |
| Dietary motivations | This theme is defined as the catalyst for changing one’s dietary behaviours and is known to play a large role in food choice decisions (4). Dietary motivations can vary largely across different diet types, with the three main motivations to the exclusion of meat and/or animal products being animal welfare, health, and environmental reasons (5). It has been poised that those who follow a vegetarian or vegan diet for health reasons may be more susceptible to diet culture. | *“I never liked meat as a kid. So, at the age of 16 I was thinking I don't like the idea of eating animals.”* – Vegetarian  *“It definitely depends on the reason why the person chose to eat a vegan diet in the first place. If it was for health reasons, it would benefit them in terms of being more conscious of what they're eating.”* – Vegan with lived eating disorder experience  *“The end goal was always vegan, just because I wanted to try to maximise the reduction of animal suffering. And so, I started off vegetarian for about five months.”* - Vegan |
| Cognitive restraint | Defined as the purposeful restriction of food intake in order to control body weight and/or shape (6). Cognitive restraint is considered to be a core feature of eating disorder pathology (2). | *“I guess people's motivations for restriction does come from different places. What's the reason for restraint? It could be because they've got a calorie limit and that's what they focus on probably majority of the time because it's easy to control.”* – Psychologist  *“In some way, we're usually or often always displaying self-control around certain foods depending on depends how habituated you are to a vegan diet. Especially if you're starting out, I think you're always like, in some stage of incongruence or having difficulty with desires and stuff. I think there are probably many vegans who also have trouble with self-control, just because sometimes other non-vegan food is tasty.”* - Vegan |
| Body dissatisfaction | Body dissatisfaction is defined as the negative attitude held towards one’s own physical appearance and thought to arise from discrepancies between actual and ideal body image (7). Body dissatisfaction is considered to be a core feature of eating disorder pathology (8). | *“I viewed myself as unattractive. I was always the chunkier one as a kid, but looking back now, I wasn't. That's just how I always perceived myself. I had that negative context in my head about it.”* – Vegetarian with lived eating disorder experience |
| Compensatory behaviours | This theme refers to behaviours intended to counteract feelings of guilt associated with eating and/or to avoid weight gain. Behaviours can include self-induced vomiting, driven exercise, fasting, or laxative or diuretic misuse (9). Body dissatisfaction is considered to be a core feature of eating disorder pathology (8). | *“Sometimes you see purging behaviours when people have quite severe anxiety or stress.”* – Dietitian  *“I wonder if someone with something like orthorexia nervosa might not even see their [exercising] behavior as compulsive because I see it as a healthy thing for themselves.”* - Dietitian |
| Vegetarian and vegan nutritional intake | Refers to the adapted nutritional requirements required to follow a vegetarian or vegan diet. For many, adherence to a vegetarian or vegan diet requires additional forethought and planning, which may contribute disordered eating thoughts. This theme also relates to potential overinflated expected positive health outcomes associated with following a vegetarian or vegan diet. | *“I don't eat a lot of those like fake meats. They just seem pointless to me. But also, the more something goes through a machine, the less of the micronutrients are going to be in there.” –* Vegetarian  *“From personal experience, I was so conscious of getting the right nutrients and meeting my needs whilst following a vegan diet. I was then so obsessed with the idea of what I was eating. I think that's where my [eating disorder] developed because it started from a good place. I was trying to be as healthy as possible. Food was just constantly in my mind because of [my vegan diet] and I just became overly obsessed and conscious of what I was eating.”* – Vegan with lived eating disorder experience |
| Preoccupation with dietary adherence | This theme is defined as a pathological obsession with one’s vegetarian or vegan dietary status, which may be related to having a higher moral virtue than omnivore diets. Those who engage in controlling thoughts about their dietary status may experience anxiety if their dietary adherence rules are broken. | *“Being in control of what you eat isn't necessarily an eating disorder. So, if you are controlling the amount of animal-based products that you're consuming, then it's not necessarily a bad thing.” –* Vegetarian  *“I'm always struck by people who are vegetarian, and then stop just suddenly and start eating meat; that baffles me.”* – Vegetarian  *“I relate because I did go through that when I was anorexic. I mean, it became an obsession again. I think it was the veganism in a way; like it became an obsession.”* – Vegan with lived eating disorder experience |

**References**

1. Lydecker JA, Simpson L, Smith SR, White MA, Grilo CM. Preoccupation in bulimia nervosa, binge-eating disorder, anorexia nervosa, and higher weight. International Journal of Eating Disorders. 2022;55(1):76-84.

2. American Psychiatric Association. Diagnostic and statistical manual of mental disorders. 5th ed. Arlington, VA2013.

3. Rosenfeld DL. Why some choose the vegetarian option: Are all ethical motivations the same? Motivation and Emotion. 2019;43(3):400-11.

4. Marty L, Chambaron S, de Lauzon-Guillain B, Nicklaus S. The motivational roots of sustainable diets: Analysis of food choice motives associated to health, environmental and socio-cultural aspects of diet sustainability in a sample of French adults. Cleaner and Responsible Consumption. 2022;5:100059.

5. North M, Klas A, Ling M, Kothe E. A qualitative examination of the motivations behind vegan, vegetarian, and omnivore diets in an Australian population. Appetite. 2021;167.

6. Stunkard AJ, Messick S. The three-factor eating questionnaire to measure dietary restraint, disinhibition and hunger. Journal of Psychosomatic Research. 1985;29(1):71-83.

7. Brechan I, Kvalem IL. Relationship between body dissatisfaction and disordered eating: Mediating role of self-esteem and depression. Eating Behaviors. 2015;17:49-58.

8. Liskov TP, Gay LJ, Fairchild MM. Vegetarianism as a potential indicator of eating disorders among dietetic internship graduates. Journal of the American Dietetic Association. 1996;96(9).

9. Colleen Stiles-Shields E, Labuschagne Z, Goldschmidt AB, Doyle AC, Le Grange D. The use of multiple methods of compensatory behaviors as an indicator of eating disorder severity in treatment-seeking youth. International Journal of Eating Disorders. 2012;45(5):704-10.
