# Supplementary material for: Development and preliminary validation of a novel eating disorder screening tool for vegetarians and vegans: the V-EDS
Source: J Eat Disord. 2024 Jan 9;12:4. doi: 10.1186/s40337-024-00964-7 (PMC10775595; doi:10.1186/s40337-024-00964-7)
Supplement: Supplementary file 2 — Additional file 2. Vegetarian Vegan Eating Disorder Screener (V-EDS). [file 40337_2024_964_MOESM2_ESM.docx]

**Additional File 2**

**Vegetarian Vegan Eating Disorder Screener (V-EDS)**

The V-EDS is a patient administered screening tool designed to assess the eating disorder symptomology in individuals following a vegetarian or vegan diet. The V-EDS has been validated in non-clinical community samples aged 18 years and over. The V-EDS is not a diagnostic tool but rather can be used to indicate whether further assessment for eating disorder symptomology may be required. Do not use the V-EDS as a substitute for professional advice. Item 1-6 do not indicate eating disorder pathology when considered in isolation, however they can be used by healthcare professionals to start a conversation around the respondents eating behaviours and attitudes.

| 1. I’m motivated to eat my food choices for: | |  |  |  |  |  |
| --- | --- | --- | --- | --- | --- | --- |
| Animal welfare | The environment | | Taste, texture, and/or smell preferences | | | |
| My health | My spiritual beliefs | | Other: ____________________________ | | | |
| **To what extent do you agree with the following statements?** | | **Strongly disagree** | **Disagree** | **Neither agree or disagree** | **Agree** | **Strongly agree** |
| 1. Your vegetarian/vegan diet is part of your identity | | 0 | 1 | 2 | 3 | 4 |
| 1. A balanced diet can include eating processed plant-based products (eg. mock meats). | | 0 | 1 | 2 | 3 | 4 |
| 1. You are willing to introduce meat to your diet if it is vital for your survival. | | 0 | 1 | 2 | 3 | 4 |
| 1. The thought of accidentally eating meat causes you significant distress. | | 0 | 1 | 2 | 3 | 4 |
| 1. Removing meat and/or animal products from your diet allows you to control the way your body looks. | | 0 | 1 | 2 | 3 | 4 |
| **On how many of the past seven days…** | | **No days** | **1-2 days** | **3-4 days** | **5-6 days** | **Every day** |
| 1. Have you experienced distress if you did not know the calorie content of food you have eaten? | | 0 | 1 | 2 | 3 | 4 |
| 1. Has thinking about the calorie content of food made it hard to think about other important things (eg, work or study)? | | 0 | 1 | 2 | 3 | 4 |
| 1. Has the way you thought about food become intrusive? | | 0 | 1 | 2 | 3 | 4 |
| 1. Have you spent a great deal of time thinking about the calorie content of food to change the way your body looks? | | 0 | 1 | 2 | 3 | 4 |
| 1. Have you eaten foods that are low in calories to control your weight or shape? | | 0 | 1 | 2 | 3 | 4 |
| 1. Have you experienced significant distress after eating? | | 0 | 1 | 2 | 3 | 4 |
| 1. Have you excluded large amounts of food to change the way your body looks? | | 0 | 1 | 2 | 3 | 4 |
| 1. Have you exercised in a compulsive manner to earn food to eat? | | 0 | 1 | 2 | 3 | 4 |
| 1. Have you experienced significant distress after eating foods that are high in calories? | | 0 | 1 | 2 | 3 | 4 |
| 1. Have you felt scared of over eating and the impact it could have on your weight or shape? | | 0 | 1 | 2 | 3 | 4 |
| 1. Have you been fearful of being fat? | | 0 | 1 | 2 | 3 | 4 |
| 1. Has it been hard for you to focus on much else than your weight or shape? | | 0 | 1 | 2 | 3 | 4 |

What is your current weight (please give your best approximate)?: __________________

What is your height (please give your best approximate)?: __________________
